# Interaction between *CD244* and *SHP2* regulates inflammation in chronic obstructive pulmonary disease via targeting the MAPK/NF-κB signaling pathway

**DOI:** 10.1371/journal.pone.0312228

**Published:** 2024-10-18

**Authors:** Xiaobing Gao, Suhua Shao, Xi Zhang, Changjie Li, Qianqian Jiang, Bo Li

**Affiliations:** 1 Department of Emergency Medicine, General Hospital of Central Theater Command, Wuhan, Hubei Province, China; 2 Department of Outpatient, General Hospital of Central Theater Command, Wuhan, Hubei Province, China; 3 Laboratory of Shanghai Yijian Medical Testing Institute, Shanghai, China; 4 Health Management Center, Renmin Hospital of Wuhan University, Wuhan, Hubei Province, China; University of Manitoba, CANADA

## Abstract

This study delved into the interplay between *CD244* and *Src Homology 2 Domain Containing Phosphatase-2* (*SHP2*) in chronic obstructive pulmonary disease (COPD) pathogenesis, focusing on apoptosis and inflammation in cigarette smoke extract (CSE)-treated human bronchial epithelial (HBE) cells. Analysis of the GSE100153 dataset identified 290 up-regulated and 344 down-regulated differentially expressed genes (DEGs). Weighted gene co-expression network analysis (WGCNA) highlighted the turquoise module had the highest correlation with COPD samples. Functional enrichment analysis linked these DEGs to critical COPD processes and pathways like neutrophil degranulation, protein kinase B activity, and diabetic cardiomyopathy. Observations on CD244 expression revealed its upregulation with increasing CSE concentrations, suggesting a dose-dependent relationship with inflammatory cytokines (IL-6, IL-8, TNF-α). CD244 knockdown mitigated CSE-induced apoptosis and inflammation, while overexpression exacerbated these responses. Co-immunoprecipitation (Co-IP) confirmed the physical interaction between CD244 and SHP2, emphasizing their regulatory connection. Analysis of Concurrently, the Nuclear Factor-kappa B (NF-κB) and Mitogen-activated protein kinase (MAPK) signaling pathways showed that modulating CD244 expression impacted key pathway components (p-JNK, p-IKKβ, p-ERK, p-P38, p-lkBα, p-P65), an effect reversed upon SHP2 knockdown. These findings underscore the pivotal role of the CD244/SHP2 axis in regulating inflammatory and apoptotic responses in CSE-exposed HBE cells, suggesting its potential as a therapeutic target in COPD treatment strategies.

## Introduction

There is a progressive respiratory disease called COPD that is characterized by persistent and often irreversible limitation of airflow [1]. It includes chronic bronchitis and emphysema, mainly caused by long-term exposure to noxious gases or particles, most commonly tobacco smoke [2]. Smoking remains the primary danger factor for COPD, accounting for the majority of cases [3]. Nonetheless, the disease also impacts a significant number of non-smokers, particularly in regions burdened by elevated levels of environmental pollution. Genetic vulnerability and environmental risk factors interact intricately in the multifactorial pathophysiology of COPD [4]. The onset and course of illness are also significantly influenced by additional variables, including genetic predisposition, indoor air pollution, and occupational exposure to dust and chemicals [5]. At present, the objectives of COPD treatment include symptom relief, enhancement of lung function, and enhancement of the overall quality of life for patients [6]. Pharmacological interventions, including bronchodilators, corticosteroids, and phosphodiesterase-4 inhibitors, are prescribed based on disease severity [7]. Pulmonary rehabilitation and supplemental oxygen therapy are additional components of the treatment approach [8]. Despite these treatment options, the long-term prognosis of COPD remains poor, with disease progression leading to increased disability and frequent exacerbations. Given the significant impact of COPD on global health, there is a pressing requirement to develop new biomarkers for diagnosis, innovative treatment modalities, and reliable prognostic factors.

Natural killer (NK) cell receptor 2B4, or *CD244*, is a cell surface receptor that is mostly expressed on CD8^+^ T lymphocytes and NK cells [9]. Research in recent years has revealed the relationship between *CD244* and various diseases. For example, a study by Hsu CL et al noted higher *CD244* expression in patients with advanced hepatocellular carcinoma who responded to immune checkpoint inhibitors [10]. Ishihara M et al. found that a large proportion of T cells generated by patients with cytokine release syndrome (CRS) expressed high levels of *CD244* [11]. According to different research, several autoimmune conditions such as rheumatoid arthritis (RA) and systemic lupus erythematosus (SLE) have different amounts of *CD244* expression [12]. This indicates that increased expression of *CD244* may be associated with disease activity and reflects the state of immune system activation. In inflammatory bowel disease (IBD), the interaction between *CD244* and its ligands may promote the inflammatory response [13]. In addition, studies have found that *CD244* binds to the inhibitory phosphatase *SHP2* or *Src Homology 2 Domain Containing Phosphatase-1* (*SHP1)* and functions as an inhibitory receptor [14]. In particular, studies have shown that *SHP2* plays a key role in regulating acute cigarette smoke-mediated lung inflammatory responses [15]. This finding suggests that the interaction between *CD244* and *SHP2* may play a significant role in the inflammatory process triggered by external environmental factors. The understanding of this mechanism provides new insights into how harmful substances such as cigarette smoke trigger inflammation in the lungs and could serve as a theoretical foundation for the development of novel therapeutic strategies targeting this type of inflammation.

MAPK signaling pathway regulates cellular responses to various stimuli, including growth factors, stress, and inflammation [16]. Activation of MAPK cascades involves sequential phosphorylation of kinases, ultimately leading to modulation of gene expression and cellular functions like cell growth, specialization, and programmed cell death [17]. Concurrently, the NF-κB signaling pathway emerges as a cardinal regulator of immune and inflammatory responses [18]. In its quiescent state, NF-κB resides in the cytoplasm, bound to inhibitory proteins [19]. Activation by stimuli, including cytokines and pathogens, prompts its nuclear translocation, initiating the transcription of genes pivotal for inflammation, immunity, and cell survival [20]. The MAPK signaling pathway can promote the activation of NF-κB, and the NF-κB signaling pathway can also affect the activity of MAPK. Studies have proven that the interaction of these two pathways has a significant part in biological processes such as inflammation, apoptosis, and immune response. Zhao Z et al. found that sodium butyrate attenuates COPD inflammation induced by cigarette smoke by activating *G Protein-Coupled Receptor 43* (*GPR43*), thereby inhibiting NF-κB/MAPK signaling pathways [21]. Similarly, Watanabe T et al. elucidated the synergistic activation of NF-κB by nontypeable Haemophilus influenzae and TNF-α in COPD, implicating distinct MAPK-involved signaling pathways in inflammation regulation [22]. Additionally, Wang W et al. revealed that carbocisteine mitigates hydrogen peroxide-induced inflammation in A549 cells by targeting the NF-κB and ERK1/2 MAPK pathways, offering insights into its potential therapeutic mechanism in COPD [23]. Furthermore, Ryu HW et al. found that Daphnodorin C from Daphne kiusiana suppresses COPD-related inflammation by inhibiting NF-κB and specific MAPK signaling pathways *in vitro* and *in vivo* [24]. These findings emphasize the imperative to elucidate the NF-κB and MAPK signaling pathways, providing a foundation for exploring therapeutic strategies for COPD.

Considering the multifaceted nature of the pathogenesis of COPD, not only genetic and environmental factors are involved, but also complex signaling networks, such as the signaling pathways of MAPK and NF-κB. This study attempted to explore the regulatory roles of *CD244* and *SHP2* in these signaling pathways in the context of COPD. Our aim was to examine the impact of varying levels of *CD244* and *SHP2* expression on the reactions of cells to exposure to cigarette smoke, such as apoptosis, inflammation, and the initiation of NF-κB and MAPK signaling pathways. COPD is a complex disease characterised by airway inflammation and epithelial damage, and chronic smoking is a major risk factor [25]. CSE-treated HBE cell models have been widely used to mimic the pathological environment of COPD because of their ability to replicate key features of the disease including oxidative stress, inflammation and apoptosis [26]. By studying these mechanisms, we aim to discover potential therapeutic targets that can mitigate the harmful effects of cigarette smoke on lung epithelial cells, ultimately helping to develop new strategies to prevent and treat COPD.

## Materials and methods

### Download of GSE100153 dataset and identification of differentially expressed genes (DEGs)

The COPD-related microarray dataset (GSE100153) was downloaded from Gene Expression Omnibus (GEO, https://www.ncbi.nlm.nih.gov/gds/) and preprocessed using R packages. The dataset included COPD samples (n = 19) and their corresponding controls (n = 24). The mean value of the expression of those probe sets was estimated in the case of several probe sets that matched the same gene. After converting the probe IDs into gene symbols, differential analysis was completed by the Limma package of the R language. The threshold criterion for fold change (FC) used was set to > 1.3 (up-regulated DEGs) or < 0.77 (down-regulated DEGs), with *p* < 0.05. Additionally, the ggplot2 package of R language was used to picture the identification results of DEGs.

### Weighted gene co-expression network analysis (WGCNA)

A comprehensive analysis of DEGs in the GSE100153 dataset was performed using the WGCNA method. A gene co-expression network was created using the R language’s "WGCNA" package. The soft threshold power is precisely tuned at β = 7 to guarantee scale-free topology. A topological overlap matrix (TOM) is created by converting the weighted adjacency matrix, which serves as a robust measure of network connectedness after the network has been built. Hierarchical clustering applied to the TOM yielded a dendrogram, wherein individual branches, depicted in varied colors, signify distinct gene modules. Using weighted correlation coefficients, DEGs exhibiting similar expression trajectories were merged into corresponding modules. Finally, the correlation between different gene modules and GSE100153 dataset samples was analyzed to determine the key module.

### Key module gene functional enrichment study as well as protein-protein interaction (PPI) network analysis

The DAVID database (https://david.ncifcrf.gov/tools.jsp) is a widely used bioinformatics tool that provides functional annotations for gene lists derived from high-throughput experiments [27]. Using the DAVID tool, we ran Gene Ontology (GO) and Kyoto Encyclopedia of Genes and Genomes (KEGG) pathway enrichment analyses on the main module of the GSE100153 dataset that was identified by WGCNA. Results were visualized using bubble plots representing rich biological processes (BP), molecular function (MF), cellular component (CC), and KEGG pathways. For the Protein-Protein Interaction (PPI) network analysis, the Search Tool for the Retrieval of Interacting Genes/Proteins (STRING; https://string-db.org/) database was employed. To create a PPI network, the genes in the key module were entered into the STRING database, delineating potential functional relationships among the proteins these genes encode. To identify important central genes in the PPI network, we applied the Maximum Cluster Centrality (MCC), Degree, and Edge Penetrating Components (EPC) algorithms using the Cytoscape plugin. The resulting genes and their interactions were then visualized using Cytoscape. The bioinformatics platform (https://bioinformatics.psb.ugent.be/webtools/Venn/) was used to perform cross-analysis on the genes in the MCC, Degree, and EPC modules to obtain overlapping genes. Lastly, the expression of eight overlapping genes in COPD and control samples was compared in the GSE100153 dataset. Data processing and visualization were carried out utilizing the "ggplot2" software. A *p*-value less than 0.05 indicated that the data obtained were statistically significant.

### Cell culture

HBE (Wuhan, China). They were raised in a humidified incubator at 37°C and 5% CO_2_ in Dulbecco’s modified Eagle’s medium (DMEM, Thermo Fisher Scientific), supplemented with 10% fetal bovine serum (FBS, Gibco), and 1% penicillin. Streptomycin (Gibco). Cells were seeded in appropriate Petri dishes and allowed to adhere overnight before processing.

### Cell treatment

The primary cause of chronic obstructive pulmonary disease is cigarette smoke (CS), which contains a lot of reactive substances [28]. To simulate the airway environment of COPD patients *in vitro*, we treated HBE cells with CSE.CSE was prepared by passing burning cigarette smoke through DMEM medium containing 10% FBS. After seeding the cells into 6-well plates during blowing, CSE concentrations of 2.5%, 5% and 10% were selected to treat the HBE cells for 24 hours to simulate the effects of different levels of smoking exposure. MAPK inhibitor SB203580 (Abcam), treatment concentration 10 μM, treatment duration 1 hour.

### Cell transfection

For transient transfection, A density of 2×10^5^ cells per well was used to seed HBE cells in 24-well plates. Transfection of the plasmid encoding *CD244* into HBE cells using an appropriate transfection method will allow the transfected cells to express the *CD244* protein for a specific period of time to achieve overexpression. Then, two specific small interfering RNAs (siRNAs) targeting *CD244* and *SHP2* was transfected into HBE cells to achieve knockdown of *CD244* and *SHP2* expression, and cells were incubated for a specific time to allow efficient knockdown of *CD244* and *SHP2*. Control: cells without any treatment, used as a reference point for experiments, used to compare the differences between experimental and control groups. si-NC (siRNA Negative Control): cells not transfected with any siRNA, or cells transfected with a non-specific siRNA, used to assess the potential impact of the transfection process on the experimental results. over-NC (Overexpression Negative Control): cells transfected with empty vector, used to assess the effect of vector itself on cell function and protein expression. As directed by the manufacturer, cells were transfected using Lipofectamine 3000 (Invitrogen, USA). To ensure the accuracy and reproducibility of the data, at least three and more independent biological replicates of all experiments were performed in this study.

### Quantitative real-time polymerase chain reaction (qRT-PCR)

Following the manufacturer’s instructions, the TRIzol reagent (Tiangen, Beijing, China) was used to extract the total RNA of HBE cells. For complementary DNA (cDNA) synthesis, we utilized a PrimeScript RT kit from Dalian, China. To do qRT-PCR with the StepOnePlus Real-Time PCR System (Applied Biosystems, Shanghai, China), SYBR Green PCR Master Mix (Takara, China) was utilized. Gene expression levels were quantified and normalized to GAPDH. All target expression levels were computed with the 2^-ΔΔCT^ technique. A set of primer sequences was found in Table 1. To ensure the accuracy and reproducibility of the data, at least three and more independent biological replicates of all experiments were performed in this study.

**Table 1:** Primer sequences for qRT-PCR.

Target	Direction	Sequence (5’-3’)
*CD244*	Forward	AACATCATATGGATATTTGCCCTGATACACCTTGAG
*CD244*	Reverse	CCAGATTATGCTGGATGCCAGGGATCAGCTGAC
*SHP2*	Forward	TATCCTCTGAACTGTGCAGATCC
*SHP2*	Reverse	TATCCTCTGAACTGTGCAGATCC
*GAPDH*	Forward	TCGACAGTCAGCCGCATCTTTT
*GAPDH*	Reverse	ACCAAATCCGTCGACCTCTT

### Western blot (WB) analysis

HBE cell protein lysates were extracted with RIPA lysis buffer (Solarbio, Beijing, China) supplemented with protease and phosphatase inhibitors (CoWin Biosciences, Nanjing, China). The BCA Protein Assay Kit was utilized to ascertain the protein content. Proteins in equal quantities were separated using 10% SDS-PAGE and then put onto PVDF membranes from Beyotime in Beijing, China. Membranes were blocked using 5% skim milk, followed by incubation with primary antibodies targeting CD244, Bax, SHP2, JNK/p-JNK, P65/p-P65, ERK/p-ERK, P38/p-P38, IKBα/p- IKBα, IKKβ/p- IKKβ, and Cleaved caspase-3 at a dilution of 1:1000, and Bcl-2 at a dilution of 1:2000 (all obtained from Abcam, China). Subsequently, membranes were incubated with suitable secondary antibodies. As an internal reference, GAPDH (Abcam, China, 1:5000) was employed. An enhanced chemiluminescence (ECL) kit (Tiangen, Beijing, China) was used to observe protein bands and captured using a ChemiDoc imaging system (Bio-Rad, Shanghai, China). To ensure the accuracy and reproducibility of the data, at least three and more independent biological replicates of all experiments were performed in this study.

### Enzyme-linked immunosorbent assay (ELISA)

Appropriately diluted cell culture supernatant samples were added to wells of ELISA plates precoated with TNF-α, IL-6, and IL-1β antibodies. After the incubation and wash steps, the enzyme-linked secondary antibody is added, followed by the chromogenic substrate. Stop the reaction and measure the absorbance at the appropriate wavelength using a microplate reader (Elabscience, China). Determine the concentrations of TNF-α, IL-6, and IL-1β by comparing the absorbance values to a standard curve generated using corresponding standards of known concentration. To ensure the accuracy and reproducibility of the data, at least three and more independent biological replicates of all experiments were performed in this study.

### Flow cytometry

For flow cytometry analysis, COPD cells were detached using trypsin-EDTA (Life Technologies Inc., Beijing, China) and washed with phosphate-buffered saline (PBS). Stain with Annexin V and propidium iodide (PI) to differentiate viable, apoptotic, and necrotic cells in compliance with the manufacturer’s guidelines. A flow cytometer (Jiyuan, Guangzhou, China) was used for the flow cytometry, and FlowJo software (FlowJo, Hangzhou, China) was used for data analysis to calculate the cell apoptosis rate. To ensure the accuracy and reproducibility of the data, at least three and more independent biological replicates of all experiments were performed in this study.

### Co-immunoprecipitation (Co-IP) assay

Examine protein-protein interactions using the Co-IP assay. Protease inhibitor-containing IP lysis solution (Thermo Fisher Scientific) was used to lyse HBE cells. Protein A/G magnetic beads were used to precipitate protein lysates after they had been treated with certain primary antibodies for an overnight period at 4°C. Perform WB analysis of immunoprecipitates as previously described. To ensure the accuracy and reproducibility of the data, at least three and more independent biological replicates of all experiments were performed in this study.

### Statistical analysis

The R programming language (version 3.6) was used to conduct the statistical analysis. Every experiment was conducted in triplicate, and the results were reported as mean ± SD. Tukey’s test was used for post-hoc analysis, and one-way ANOVA was used to evaluate the significance of differences. The statistical significance level was set at *P*<0.05.

## Results

### Identification of co-expressed modules and key genes associated with COPD using WGCNA

**Fig 1:**
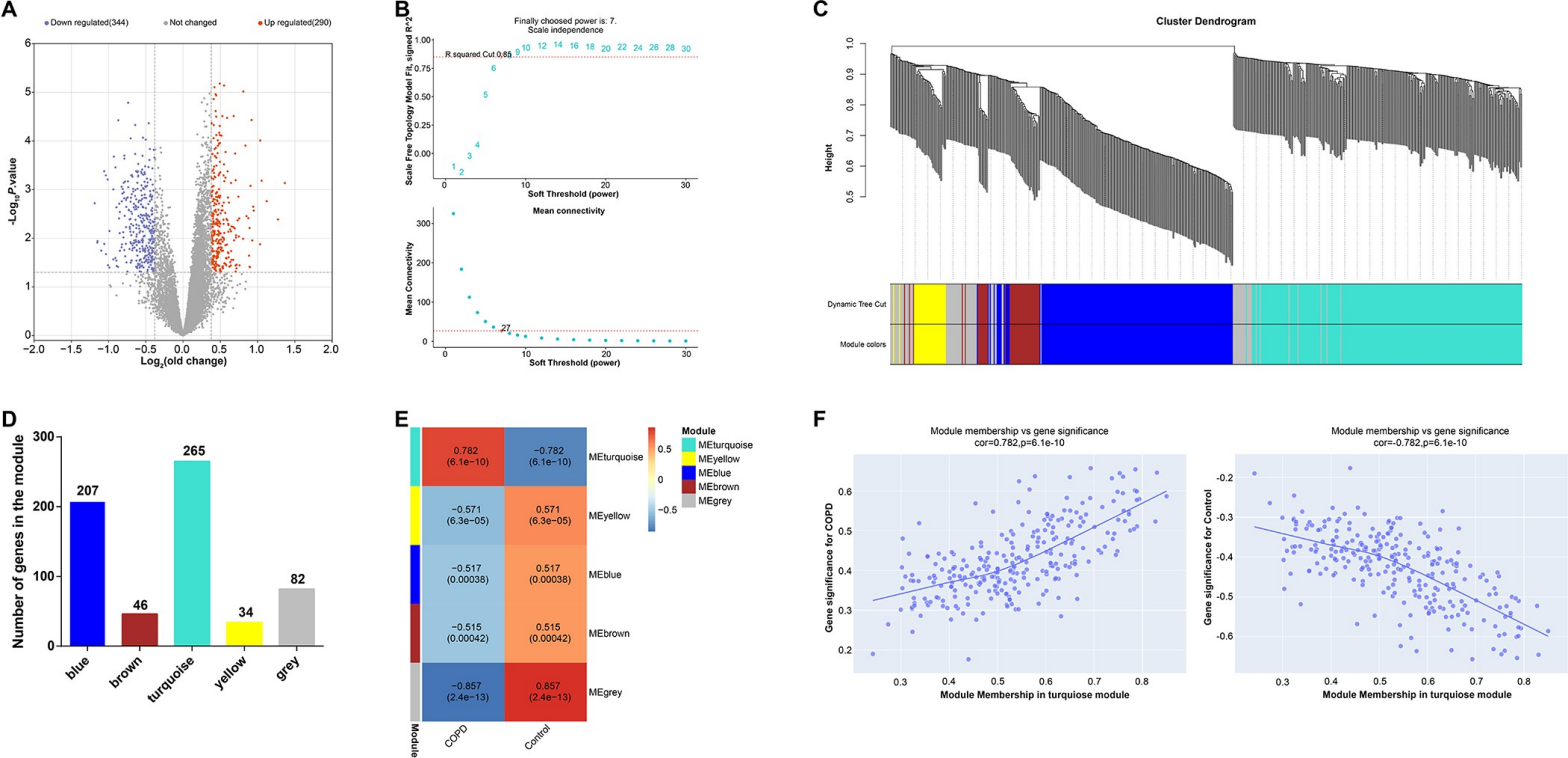
Screening of DEGs and construction of gene co-expression network in GSE100153 dataset. (A) Volcano plot showing the results of differential gene expression analysis, where red dots represent upregulated DEGs and blue dots represent downregulated DEGs. (B) Analysis of the scale-free topology fit index (β) to select an appropriate soft threshold (power value) to construct the co-expression network. (C) Dendrogram of genes clustered according to co-expression patterns, different colors represent different co-expression modules. (D) Distribution of genes in modules identified in WGCNA, showing the number of genes in each module. (E) Heatmap of correlations between co-expressed modules and clinical features. Each cell contains the corresponding correlation and *p*-value. (F) Scatterplot of GS versus MM in the turquoise module. DEGs: Differentially expressed genes; WGCNA: Weighted gene co-expression network analysis; GS: Gene significance; MM: Module membership.

In the GSE100153 dataset, we screened 290 DEGs upregulated and 344 DEGs downregulated (Fig 1A). To further understand the underlying gene co-expression patterns, we created a co-expression network of DEGs using the R WGCNA package. After evaluating the scale-free topology fit, we determined that a soft threshold of 7 provided the best trade-off between module connectivity and independence (Fig 1B). Subsequently, we identified five distinct co-expression modules, each represented by a unique color (Fig 1C–1E). Any unassigned genes are grouped in gray modules. Correlations between co-expressed modules and clinical features were examined (Fig 1E), and four modules showed significant associations with COPD. The turquoise module (r = 0.782, *P* = 6.1e-10) showed the strongest connection with COPD among them all. The relationship between the turquoise module and COPD samples was further illustrated by gene significance (GS) and module membership (MM) analysis (Fig 1F)

### Identification and functional characterization of key genes related to COPD pathogenesis in the turquoise module

**Fig 2:**
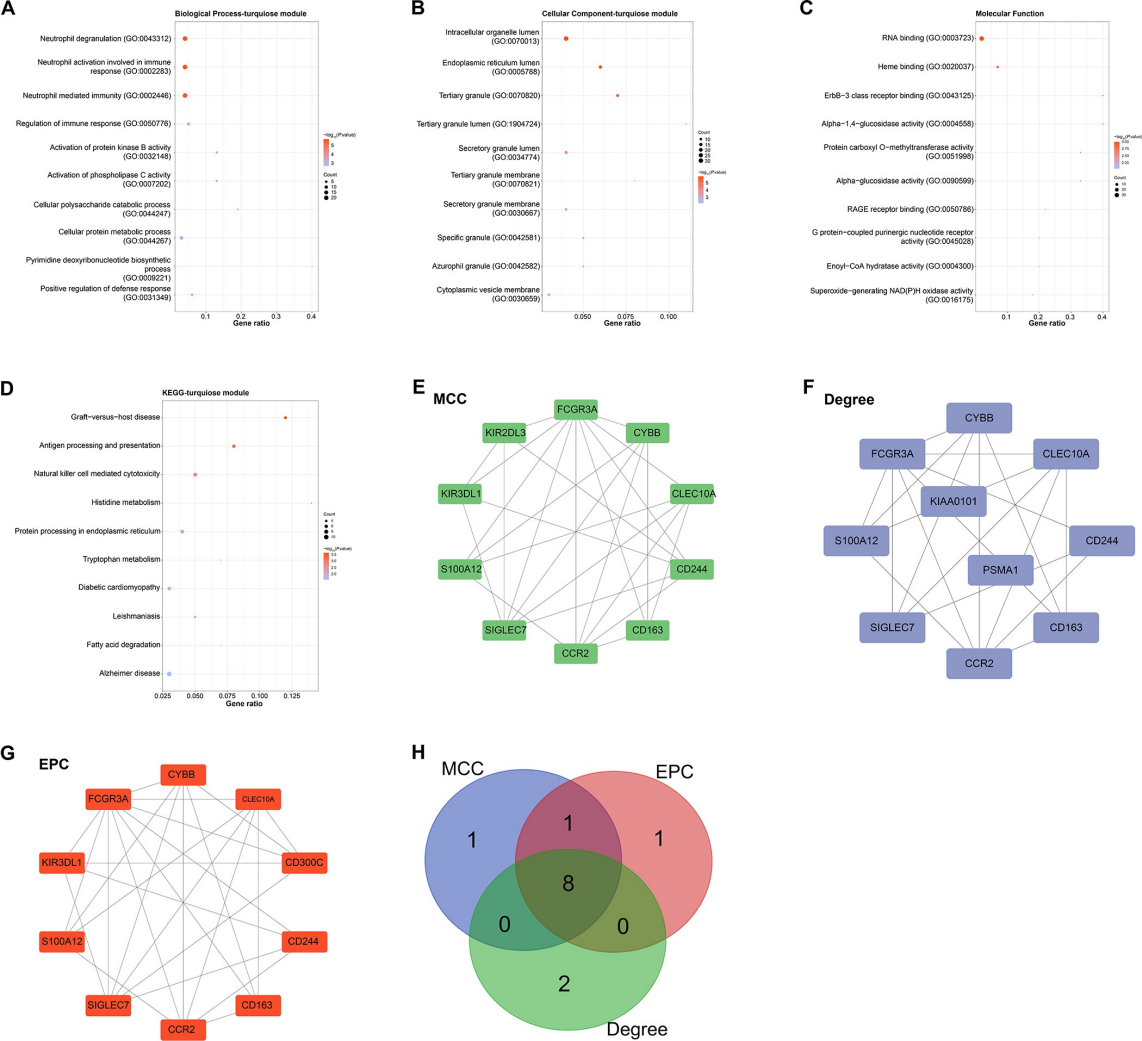
Functional enrichment and identification of overlapping genes associated with COPD in the turquoise module. (A-D) GO and KEGG pathway enrichment analysis of genes in the turquoise module. Horizontal axis (X axis): Indicates Gene Ontology terms or pathways related to BP, CC, MF, and KEGG. Vertical axis (Y-axis): -log_10_ (*P*-value) representing enriched items or enriched pathways. Each point represents a specific GO term or KEGG pathway. The size of the dots can indicate the number of genes associated with the corresponding term or pathway, with larger dots representing a greater number of genes. The color intensity of the dots can represent enrichment importance, with darker colors indicating more significant enrichments. (E-G) Topological analysis using the Cytoscape plugin to identify key genes in the turquoise module. (E) MCC analysis identified 10 nodes and 26 edges. (F) Degree analysis identified 10 nodes and 20 edges. (G) EPC analysis identified 10 nodes and 29 edges. (H) Venn diagram showing the overlap of key genes identified by three topological analyses. COPD: Chronic obstructive pulmonary disease; GO: Gene Ontology; KEGG: Kyoto Encyclopedia of Genes and Genomes; BP: Biological processes; MF: Molecular function; CC: Cellular component; MCC: Maximum Cluster Centrality; EPC: Edge Penetrating Components.

**Fig 3:**
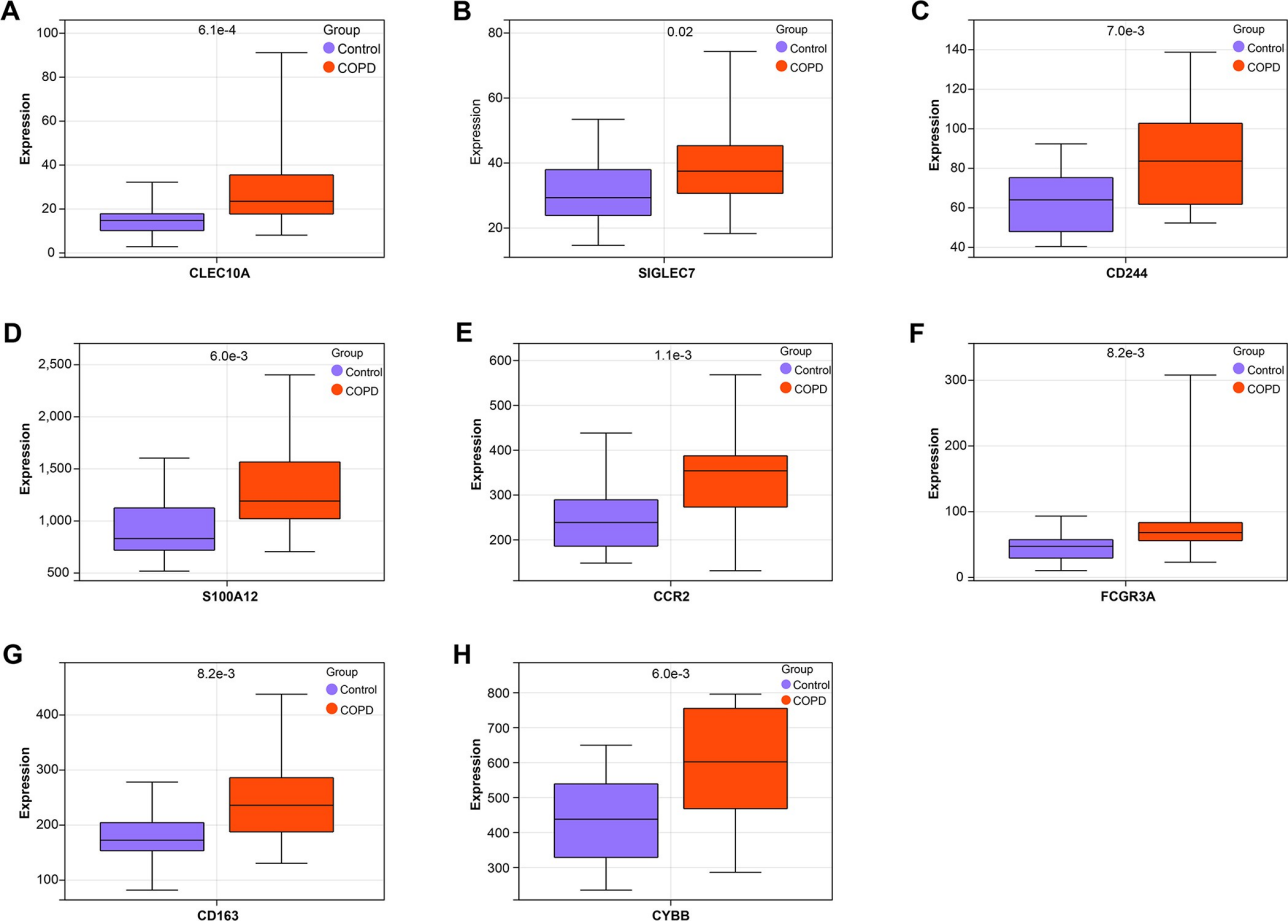
Expression analysis of overlapping genes in COPD. (A-H) Box plots of expression analysis of eight overlapping genes (*CLEC10A*, *SIGLEC7*, *CD244*, *S100A12*, *CCR2*, *FCGR3A*, *CD163*, *CYBB*) in different samples of the GSE100153 dataset. Purple represents the control group, and orange represents the COPD group. COPD: Chronic obstructive pulmonary disease.

We performed GO and KEGG enrichment analysis of genes related to the turquoise module (Fig 2A–2D). The results showed that these DEGs were enriched in neutrophil degranulation and activation of protein kinase B activity (BP), intracellular organelle lumen and tertiary granules (CC), RNA binding and heme binding (MF), etc. Furthermore, pathway analysis revealed enrichment for Diabetic cardiomyopathy, Alzheimer disease, and fatty acid degradation, implying their possible contributions to the development of COPD. To identify key genes in the turquoise module, we employed three topological analysis methods using the Cytoscape plugin, including MCC (10 nodes and 26 edges), degree (10 nodes and 20 edges), and EPC (10 nodes and 29 edges) (Fig 2E–2G). By integrating the results of these analyses, a total of eight overlapping genes were identified (Fig 2H). Further analysis results showed consistent upregulation of these eight genes (*CLEC10A*, *SIGLEC7*, *CD244*, *S100A12*, *CCR2*, *FCGR3A*, *CD163*, *CYBB*) in COPD samples from the GSE100153 dataset (Fig 3A–3H), indicating that they could be crucial in the pathogenesis of COPD. In view of this potential significance, in this study, we selected *CD244* from eight genes for further analysis to elucidate its specific role in disease mechanisms.

### CSE enhances *CD244* expression in HBE

**Fig 4:**
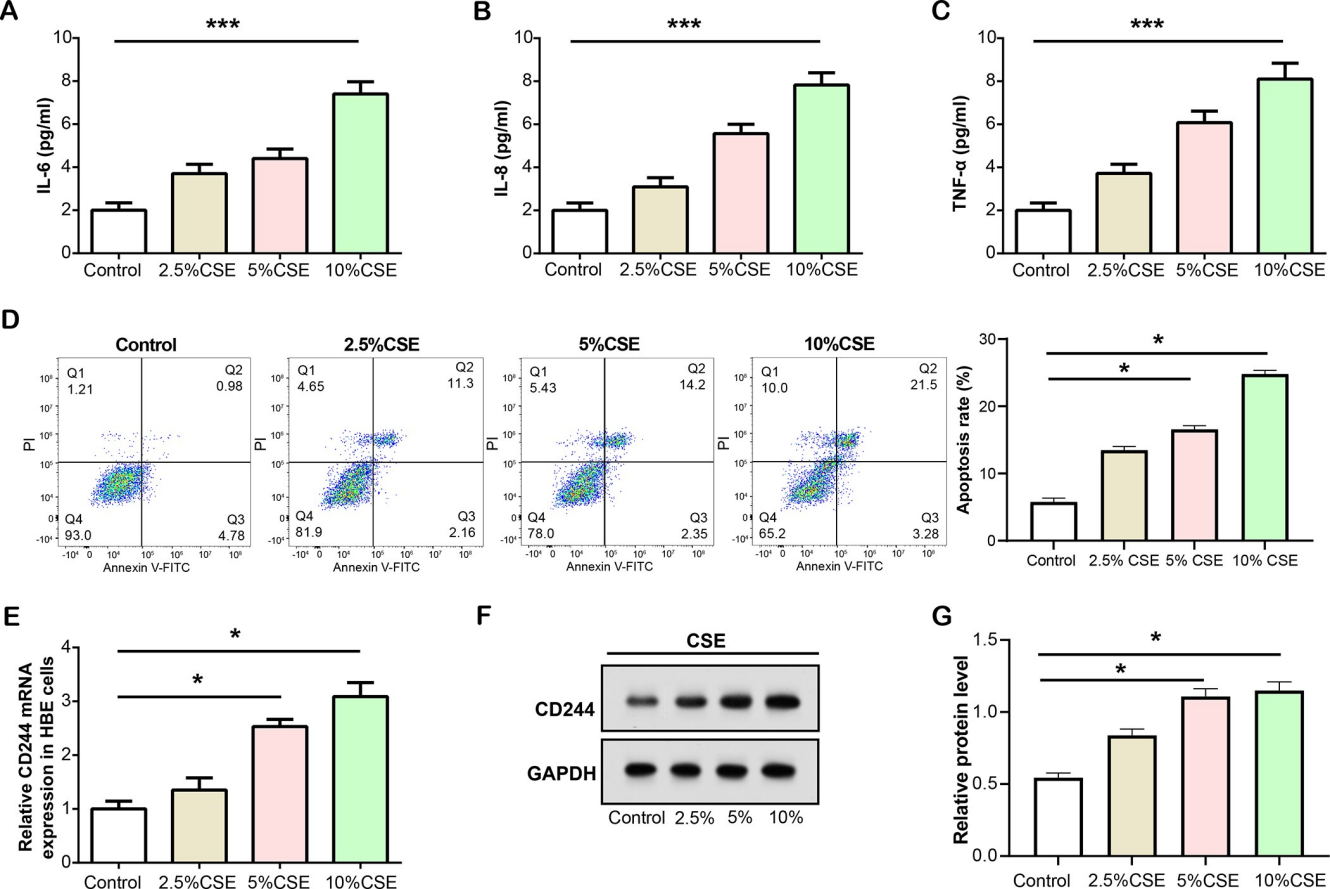
CSE enhances *CD244* expression and induces inflammation in HBE. (A-C) ELISA analysis of inflammation levels (including IL-6, IL-8, and TNF-α) in HBE cells treated with different concentrations of CSE (2.5%, 5%, and 10%). (D) Apoptosis of HBE cells treated with different concentrations of CSE (2.5%, 5% and 10%) was analysed by flow cytometry. (E) qRT-PCR analysis of *CD244* expression in HBE cells treated with different concentrations (2.5%, 5%, and 10%) of CSE. (F and G) WB analysis of *CD244* protein levels in HBE cells exposed to graded concentrations of CSE. CSE: Cigarette smoke extract; HBE: Human bronchial epithelial; ELISA: Enzyme-linked immunosorbent assay; qRT-PCR: Quantitative real-time polymerase chain reaction, WB: Western blot. *P<0.05, ***P<0.001.

We divided CSE into 2.5%, 5%, and 10% concentration gradients and treated HBE cells with each concentration for 24 hours. We then assessed the levels of inflammation, including IL-6, TNF-α, and IL-8, in CSE-treated HBE cells by ELISA (Fig 4A–4C). The results showed that as the concentration of CSE increased, the levels of inflammatory cytokines also increased significantly, indicating a concentration-dependent effect on the inflammatory response. Subsequently, we analysed the apoptosis of HBE cells that were stormed with different degrees of CSE using flow cytometry (Fig 4D). The results showed that the apoptotic rate of HBE induced by different degrees of CSE increased with the increase of CSE concentration. Next, we assessed the expression of *CD244* in HBE cells exposed to varying degrees of CSE using qRT-PCR and WB analysis (Fig 4E–4G). As the concentration of CSE increased, so did the expression of *CD244*, suggesting a dose-dependent relationship between CSE exposure and *CD244* expression in HBE cells.

### *CD244* knockdown inhibits CSE-induced apoptosis and inflammation in HBE cells

**Fig 5:**
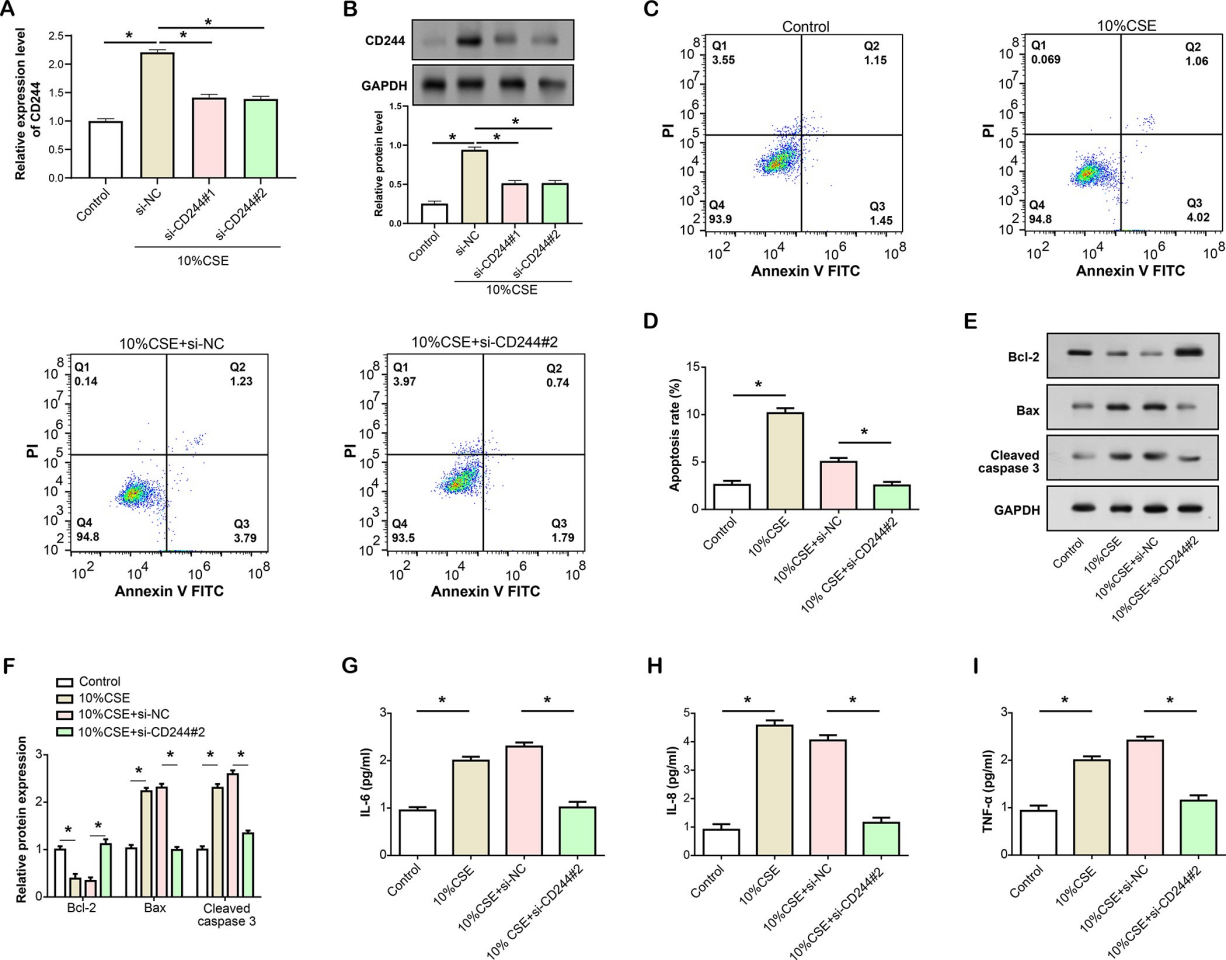
Knockdown of *CD244* inhibits CSE-induced apoptosis and inflammation in HBE cells. (A) qRT-PCR analysis showing the knockdown efficiency of *CD244* using siRNA in HBE cells induced by 10% CSE. (B) WB analysis confirmed the decrease of *CD244* protein level after *CD244* knockdown. (C and D) Flow cytometric analysis of apoptosis in HBE cells induced by 10% CSE and *CD244* knockdown. (E and F) WB analysis of apoptosis-related proteins (Bcl-2, Bax, Cleaved caspase 3) in HBE cells induced by 10% CSE and *CD244* knockdown. (G-I) ELISA analysis of expression levels of inflammatory factors (IL-6, IL-8, TNF-α) in HBE cells induced by 10% CSE and *CD244* knockdown. CSE: Cigarette smoke extract; HBE: Human bronchial epithelial; ELISA: Enzyme-linked immunosorbent assay; qRT-PCR: Quantitative real-time polymerase chain reaction, WB: Western blot. *P<0.05.

First, we evaluated the knockdown efficiency of *CD244* using qRT-PCR and WB analysis (Fig 5A and 5B). The results confirmed that *CD244* expression was significantly reduced after *CD244* knockdown, and si-*CD244*#2 showed the most obvious downregulation. Therefore, we chose si-*CD244*#2 for later trials. Next, we investigated the impact of *CD244* knockdown on CSE-induced apoptosis using flow cytometry (Fig 5C and 5D). The data showed that CSE treatment resulted in increased apoptosis rate, whereas *CD244* knockdown in CSE-treated HBE cells resulted in significantly reduced levels of apoptosis rate, similar to the control group. Subsequently, we employed WB analysis to assess the expression levels of proteins associated with apoptosis (Fig 5E and 5F). After 10% CSE exposure, Bcl-2 expression decreased, while Cleaved caspase 3 and Bax expression increased. However, when *CD244* was knocked down in CSE-treated cells, the protein expression levels of Bcl-2, Cleaved caspase 3, and Bax returned to the levels observed in the control group. Furthermore, we examined the effect of *CD244* knockdown on CSE-induced inflammation using ELISA (Fig 5G–5I). The findings demonstrated that CSE therapy raised inflammatory levels, as seen by elevated TNF-α, IL-8, and IL-6 levels. However, after the knockdown of *CD244* in CSE-treated HBE cells, the level of inflammation returned to that observed in the control group.

### *CD244* overexpression promotes CSE-induced apoptosis and inflammation in HBE cells

**Fig 6:**
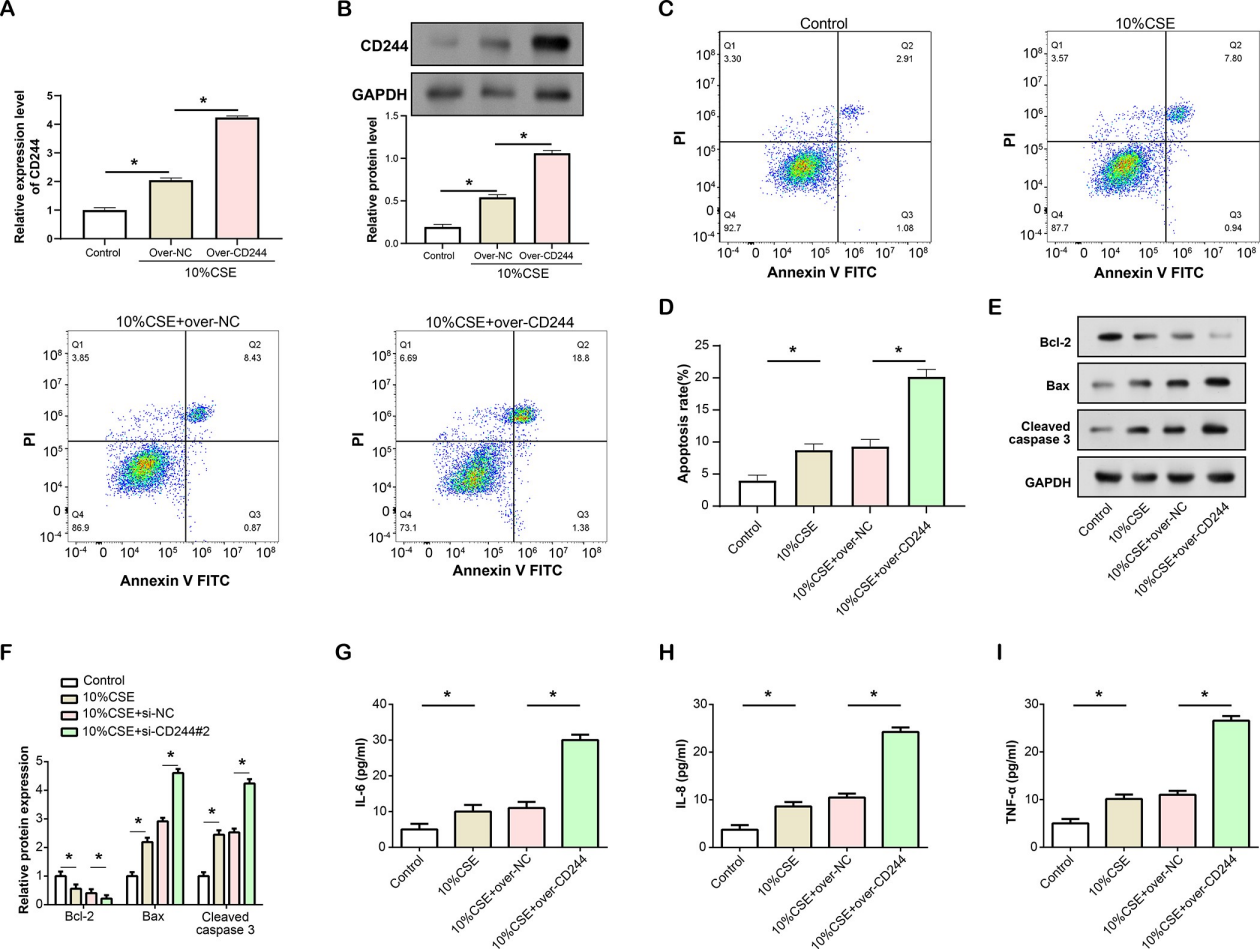
*CD244* overexpression promotes CSE-induced apoptosis and inflammation in HBE. (A) qRT-PCR analysis showing the efficiency of *CD244* overexpression in HBE cells induced by 10% CSE. (B) WB analysis confirmed a significant increase in *CD244* protein level after *CD244* overexpression, indicating successful upregulation of *CD244* in HBE cells induced by 10% CSE. (C and D) Flow cytometry analysis of apoptosis in HBE cells induced by 10% CSE and treated with *CD244* overexpression. (E and F) WB analysis of apoptosis-related proteins (Bcl-2, Bax, Cleaved caspase 3) in HBE cells induced by 10% CSE and treated with *CD244* overexpression. (G-I) ELISA analysis of inflammatory factors (IL-6, IL-8, TNF-α) levels in HBE cells induced by 10% CSE and treated with *CD244* overexpression. CSE: Cigarette smoke extract; HBE: Human bronchial epithelial; ELISA: Enzyme-linked immunosorbent assay; qRT-PCR: Quantitative real-time polymerase chain reaction, WB: Western blot. *P<0.05.

qRT-PCR and WB analyses were conducted to assess the overexpression efficiency of *CD244* (Fig 6A and 6B). The findings verified a noteworthy rise in *CD244* expression, indicating successful upregulation in HBE cells. Flow cytometry was then used to evaluate the impact of *CD244* overexpression on CSE-induced apoptosis rate (Fig 6C and 6D). Data revealed that the rate of apoptosis increased after receiving CSE therapy, with overexpression of *CD244* in CSE-treated HBE cells exacerbating this effect. Subsequently, the expression levels of apoptosis-related proteins were assessed by WB analysis (Fig 6E and 6F). Bax and Cleaved caspase 3 levels increased while Bcl-2 protein expression decreased after CSE stimulation. Notably, *CD244* overexpression in CSE-induced HBE cells resulted in a further reduction in Bcl-2 expression and a significant enhancement in Cleaved caspase 3 and Bax levels. Moreover, ELISA assays were employed to examine the effects of *CD244* overexpression on CSE-induced inflammation (Fig 6G–6I). The outcomes indicated that CSE induction led to elevated expression levels of inflammatory factors, with the combination of CSE treatment and *CD244* overexpression resulting in a more pronounced increase in inflammation levels compared to CSE treatment alone.

### Interaction between *CD244* and *SHP2* in CSE-induced HBE cells

**Fig 7:**
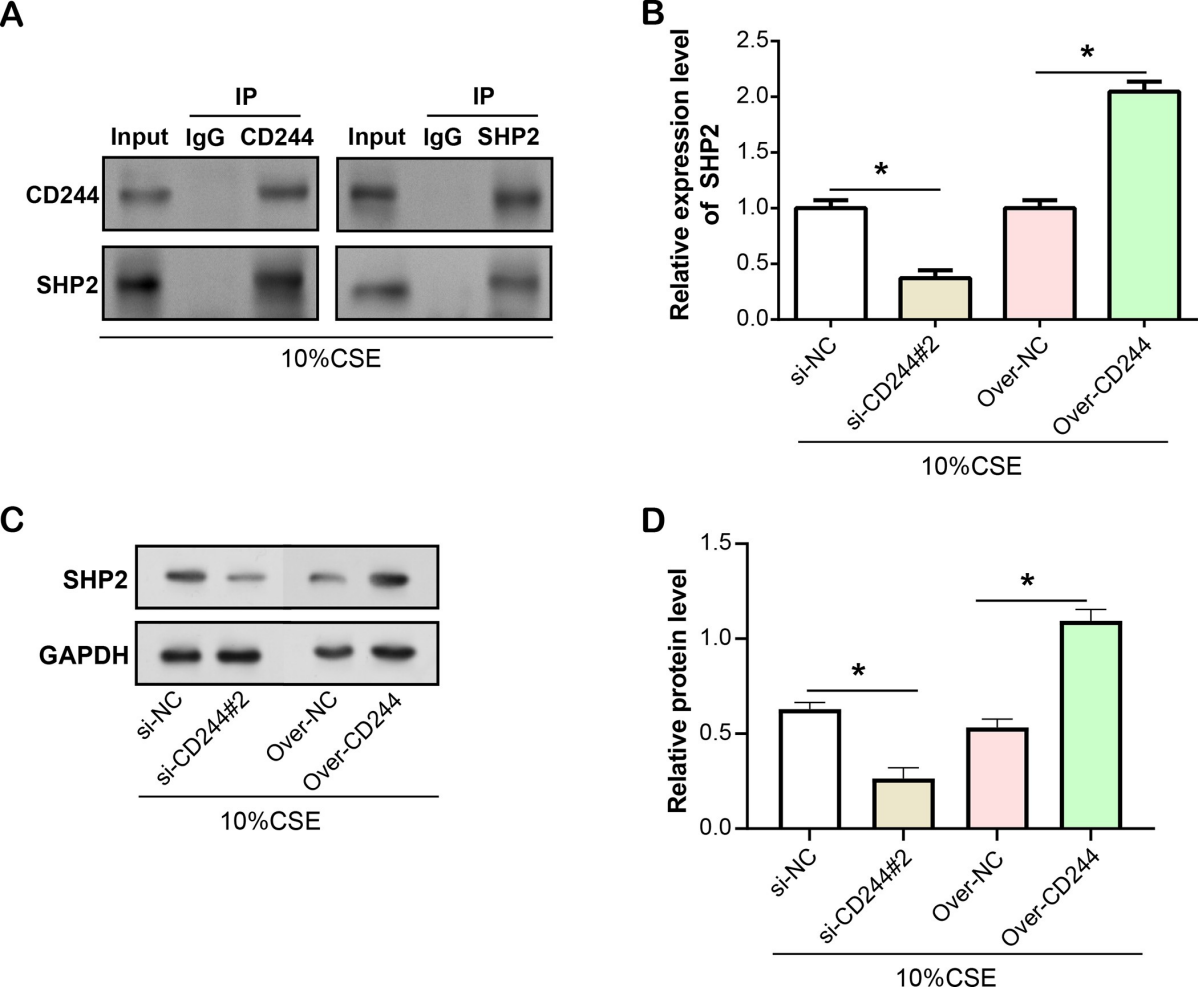
Interaction between *CD244* and *SHP2* in CSE-induced HBE. (A) Co-IP analysis confirmed the physical interaction between *CD244* and *SHP2* in HBE cells induced by 10% CSE. (B) qRT-PCR analysis of *SHP2* mRNA levels after *CD244* knockdown or overexpression in 10% CSE-induced HBE cells. (C and D) WB analysis of *SHP2* protein levels after *CD244* knockdown or overexpression in 10% CSE-induced HBE cells. CSE: Cigarette smoke extract; HBE: Human bronchial epithelial; Co-IP: Co-immunoprecipitation; qRT-PCR: Quantitative real-time polymerase chain reaction, WB: Western blot. *P<0.05.

To further elucidate the role of *CD244* in COPD, we focused on protein-protein interactions involving *CD244*. Previous data reported the involvement of *SHP2* in cigarette smoke-induced lung inflammation [29]. To explore this association, we performed Co-IP assays (Fig 7A). As expected, in the Co-IP assay against *SHP2*, *CD244* was detected in the immunoprecipitated complex, confirming their physical interaction. Subsequently, to look into the effect of *CD244* on *SHP2* expression in the presence of CSE exposure, we assessed the expression levels of *SHP2* in *CD244*-knockdown and overexpression HBE cells using qRT-PCR and WB analysis (Fig 7B–7D). The results showed that knockdown of *CD244* resulted in decreased expression of *SHP2*, whereas overexpression of *CD244* resulted in increased expression of *SHP2*. This indicates a positive correlation between *CD244* and *SHP2*.

### Overexpression of *CD244* promotes CSE-induced apoptosis and inflammation in HBE cells, which can be reversed by knockdown of *SHP2*

**Fig 8:**
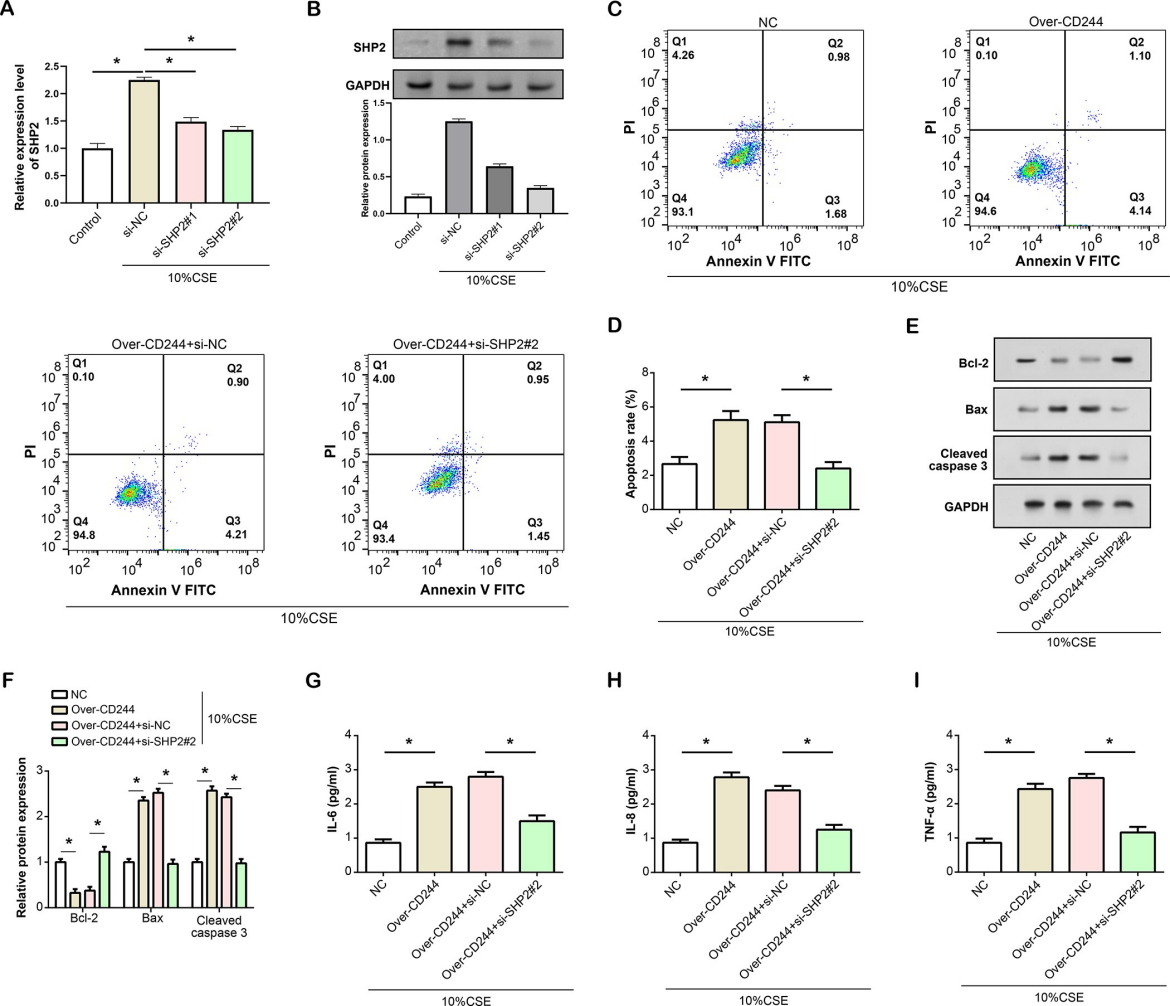
Regulatory impact of *CD244* and *SHP2* on apoptosis and inflammation in CSE- induced HBE cells. (A) qRT-PCR analysis to detect the efficiency of *SHP2* knockdown in HBE cells induced by 10% CSE. (B) WB analysis of the efficiency of *SHP2* knockdown in HBE cells induced by inflammatory 10% CSE. (C-E) Flow cytometry to detect the effect of *SHP2* knockdown on *CD244*-mediated apoptosis in HBE cells induced by 10% CSE. (F) WB analysis of the expression levels of apoptosis-related proteins (Bcl-2, Bax, Cleaved caspase 3) in HBE cells induced by 10% CSE after overexpression of *CD244* and knockdown of *SHP2*. (G-I) ELISA analysis of the effects of *SHP2* knockdown on over-*CD244*-mediated inflammatory factors (IL-6, IL-8, TNF-α) levels in HBE cells induced by 10% CSE. CSE: Cigarette smoke extract; HBE: Human bronchial epithelial; ELISA: Enzyme-linked immunosorbent assay; qRT-PCR: Quantitative real-time polymerase chain reaction, WB: Western blot. *P<0.05.

Initially, we assessed the efficiency of *SHP2* knockdown using qRT-PCR and WB analysis (Fig 8A and 8B). The results indicated a reduction in *SHP2* expression, with si-*SHP2*#2 showing the most significant downregulation. Next, we assessed the effect of *SHP2* knockdown on *CD244*-mediated apoptosis using flow cytometry (Fig 8C and 8D). The data suggest that overexpression of *CD244* in HBE cells increases apoptosis rate. However, when *SHP2* was knocked down in *CD244*-overexpressing HBE cells, the level of apoptosis was restored to that observed in the control group. Subsequently, we employed WB analysis to assess the expression levels of proteins associated with apoptosis (Fig 8E and 8F). Following the overexpression of *CD244*, Bcl-2 protein expression declined, whilst Bax and Cleaved caspase 3 expression rose. However, when *SHP2* was knocked out in *CD244*-overexpressing HBE cells, the protein expression levels of Bcl-2, Bax, and Cleaved caspase 3 returned to the levels observed in the control group. Furthermore, we examined the effect of *SHP2* knockdown on *CD244*-mediated inflammation using ELISA (Fig 8G–8I). The findings revealed that overexpression of *CD244* elevates inflammation markers. However, the concurrent knockdown of *SHP2* in *CD244*-overexpressed cells mitigated this inflammatory response. These data suggest that *CD244* and *SHP2* are key regulators of apoptosis and inflammation in the context of CSE exposure of HBE cells and that *SHP2* may counteract the pro-apoptotic and pro-inflammatory effects of *CD244*.

### Regulation of NF-κB and MAPK signaling pathway activation in CSE-treated HBE cells by *CD244* and *SHP2*

**Fig 9:**
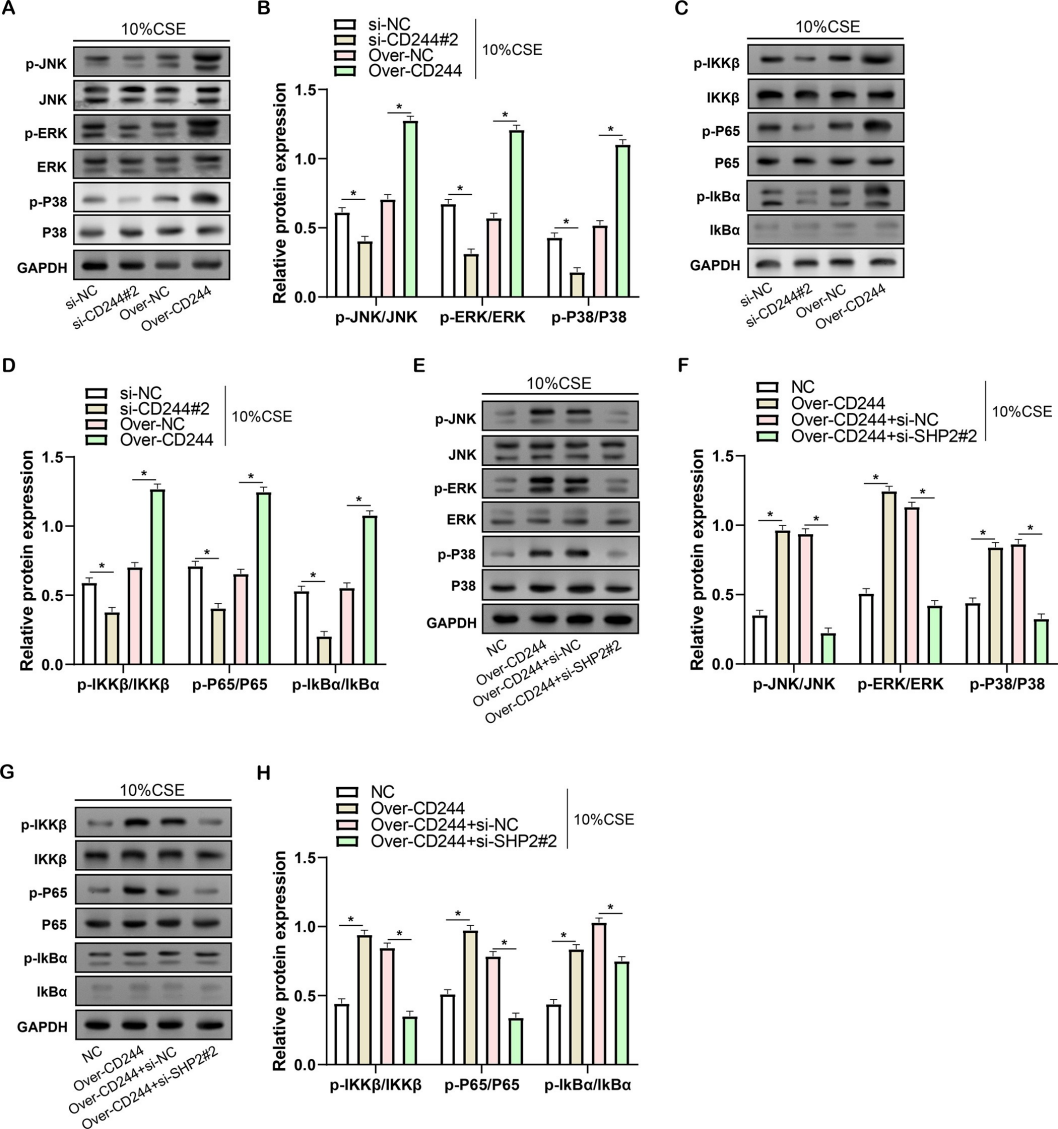
Regulation of MAPK and NF-κB signaling pathway activation by *CD244* and *SHP2* in CSE-treated HBE cells. (A and B) WB analysis of the effects of *CD244* knockdown and overexpression on MAPK signaling pathway-related protein (p-JNK/JNK, p-ERK/ERK, p-P38/P38) levels in HBE cells induced by 10% CSE. (C and D) WB analysis of the effects of *CD244* knockdown and overexpression on NF-κB signaling pathway-related protein (p-IKKβ/IKKβ, p-P65/P65, p-IκBα/IκBα) levels in HBE cells induced by 10% CSE. (E and F) WB analysis of the effects of *CD244* overexpression combined with *SHP2* knockdown on MAPK signaling pathway-related protein (p-JNK/JNK, p-ERK/ERK, p-P38/P38) levels in HBE cells induced by 10% CSE. (G and H) WB analysis of the effects of *CD244* overexpression combined with *SHP2* knockdown on NF-κB signaling pathway-related protein (p-IKKβ/IKKβ, p-P65/P65, p-IκBα/IκBα) levels in HBE cells induced by 10% CSE. CSE: Cigarette smoke extract; HBE: Human bronchial epithelial; COPD: Chronic obstructive pulmonary disease; WB: Western blot.

NF-κB and MAPK signaling pathways play extensive roles in the pathogenesis of COPD. Therefore, to investigate whether *CD244* and *SHP2* affect COPD through the MAPK and NF-κB signaling pathways, we investigated the phosphorylation status of these pathway components in HBE cells treated with CSE. Knockdown of CD244 results in decreased ratios of p-JNK/JNK, p-ERK/ERK, p-P38/P38, p-IKKβ/IKKβ, p-P65/P65 and p-lkBα/IkBα (Fig 9A–9D). In contrast, overexpression of *CD244* led to the opposite result. Furthermore, when *CD244* was overexpressed and *SHP2* was simultaneously knocked down, the ratios of p-JNK/JNK, p-ERK/ERK, p-P38/P38, p-IKKβ/IKKβ, p-P65/P65 and p-lkBα/IkBα were restored to the levels of controls (Fig 9E–9H). This suggests that *SHP2* may act as a negative regulator in these pathways, counteracting the effects of *CD244* and potentially alleviating its pro-inflammatory and pro-apoptotic activities in the context of COPD.

### SB203580 inhibitor reverses the promotional effect of *CD244* overexpression on CSE-induced apoptosis and inflammatory response in HBE cells

**Fig 10:**
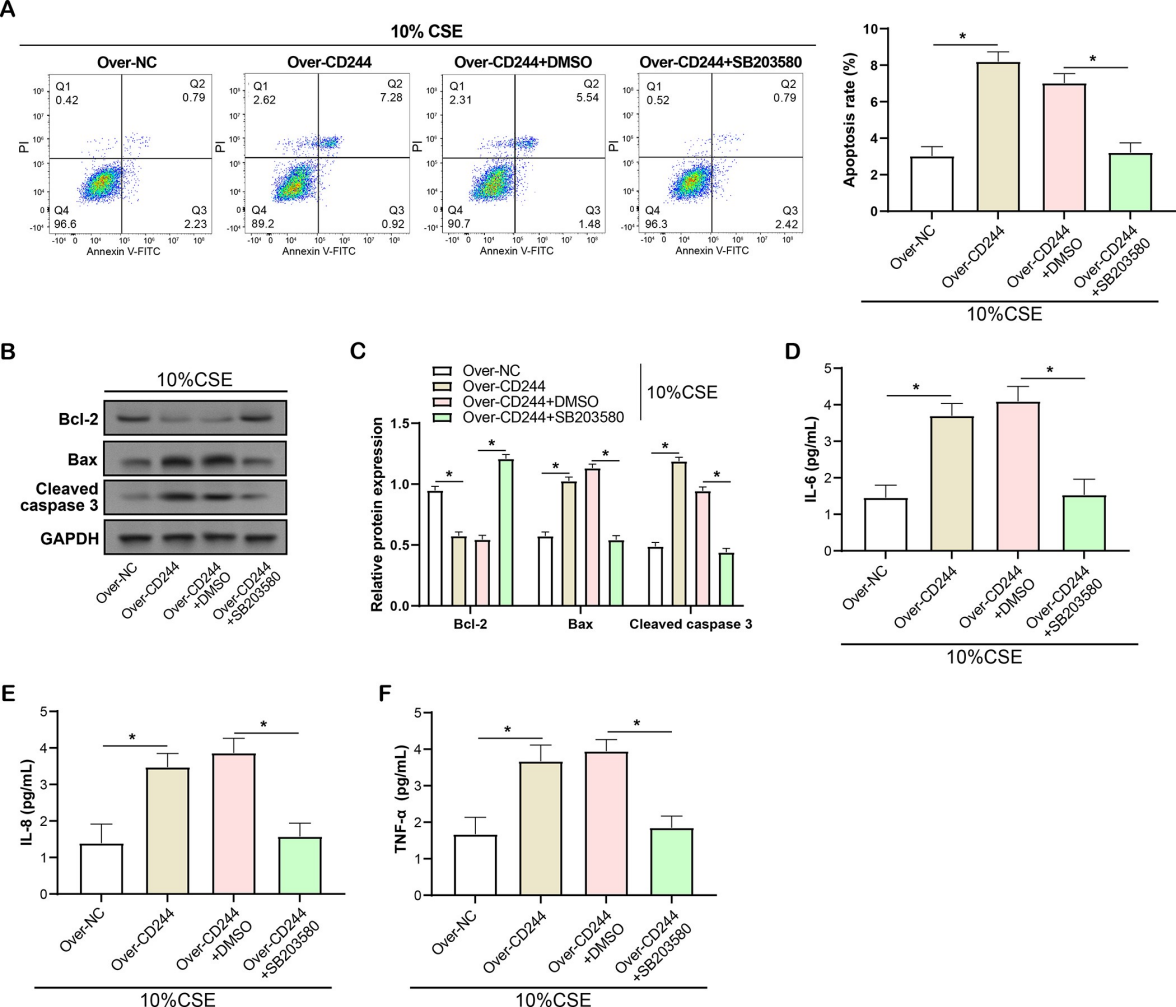
SB203580 inhibitor reverses the promotional effect of *CD244* overexpression on CSE-induced apoptosis and inflammatory response in HBE cells. (A) Flow cytometry to detect the effect of SB203580 inhibitor on *CD244*-mediated apoptosis in 10% CSE-induced HBE cells. (B and C) WB analysis of the expression levels of apoptosis-related proteins (Bcl-2, Bax, Cleaved caspase 3) in 10% CSE-induced HBE cells after overexpression of *CD244* and SB203580 inhibitor treatment. (D-F) ELISA was performed to analyse the effect of SB203580 inhibitor on the levels of per *CD244*-mediated inflammatory factors (IL-6, IL-8, TNF-α) in 10% CSE-induced HBE cells. CSE: Cigarette smoke extract; HBE: Human bronchial epithelium; WB: Immunoblot; ELISA: Enzyme-linked immunosorbent assay. *P<0.05.

To further confirm that *CD244* and *SHP2* are regulating the response of CSE-treated HBE cells through the MAPK and NF-κB signalling pathways, we employed SB203580, an inhibitor of the MAPK signalling pathway, to treat *CD244* overexpressing cells. Our study first evaluated the effect of SB203580 inhibitor on *CD244*-mediated apoptosis in 10% CSE-induced HBE cells using flow cytometry (Fig 10A). The results showed that the SB203580 inhibitor could effectively inhibit the promotion of apoptosis by *CD244* in HBE cells, reducing the apoptosis rate to a level comparable to that of the control. Next, we analysed the expression levels of apoptosis-related proteins (Bcl-2, Bax, Cleaved caspase 3) in *CD244* overexpressing HBE cells under the treatment of SB203580 inhibitor by WB (Fig 10B and 10C). The experimental data revealed that the SB203580 inhibitor was able to inhibit the regulation of the expression of apoptosis-related proteins by *CD244*, which contributed to the restoration of the expression of these proteins to the control level. In addition, we explored the effect of SB203580 inhibitor on the levels of *CD244*-mediated inflammatory factors (IL-6, IL-8, TNF-α) in 10% CSE-induced HBE cells by ELISA analysis (Fig 10D–10F). The results of the analyses showed that the SB203580 inhibitor was able to inhibit the promotional effect of *CD244* on the expression of inflammatory factors, resulting in a reduction in the levels of these factors to levels close to those of the control group. These experimental data strongly support the idea that *CD244* and SHP2 regulate apoptosis and inflammatory responses in CSE-treated HBE cells through MAPK and NF-κB signalling pathways.

## Discussion

COPD poses a significant health threat worldwide, characterized by progressive airflow limitation and presenting substantial mortality rates [30]. The current diagnostic modalities for COPD predominantly rely on clinical symptoms, spirometry, and imaging findings [31]. However, despite their utility, these methods often fall short in detecting the disease at its early stages, thereby delaying the initiation of treatment and ultimately compromising patient outcomes. In light of these challenges, there has been a burgeoning interest in investigating targeted gene therapy as a possible route to tackle the underlying pathogenesis of COPD. Our study identifies *CD244* as a hub gene, making it a promising candidate for targeted therapy due to its involvement in inflammatory processes. By targeting *CD244*, it is possible to modulate the inflammatory cascade that is a hallmark feature of COPD. The search for *CD244*-targeted therapies opens new vistas for the treatment of COPD, providing innovative strategies to address this debilitating respiratory disease.

In this work, we examined the GSE100153 dataset using bioinformatics techniques and obtained a total of 634 DEGs. WGCNA analysis of DEGs was performed and it was found that the turquoise module had the highest correlation with COPD, demonstrating its possible involvement in the etiology of disease. Further functional enrichment analysis showed that turquoise module significant gene enrichment was observed in terms or pathways such as Neutrophil degranulation, Activation of protein kinase B activity, Diabetic cardiomyopathy, RNA binding, etc. Climent M et al. discovered a relationship between diabetic cardiomyopathy and COPD influenced by microRNA and ROS crosstalk, contributing to oxidative stress in both cardiac and pulmonary diseases [32]. Similarly, Ricciardi L et al. found decreased expression of RNA binding proteins, particularly AUF-1, in the bronchial epithelium of COPD patients, potentially disrupting posttranscriptional gene regulation [33]. In addition, some studies have pointed out that the activation of neutrophil degranulation and protein kinase B activity has a major part in lung damage and disease progression in COPD by promoting inflammatory responses and affecting cell survival [34]. To prioritize key genes in the turquoise module, we applied three topological analysis methods, resulting in the identification of eight overlapping genes (*CLEC10A*, *SIGLEC7*, *CD244*, *S100A12*, *CCR2*, *FCGR3A*, *CD163*, *CYBB*). Expression analysis of these genes consistently showed their high expression in COPD, supporting their potential role as key drivers of COPD pathophysiology.

CSE is a concentrated solution obtained by bubbling smoke from burning cigarettes through a suitable solvent, usually a cell culture medium [35]. It contains a complex mixture of thousands of chemicals, including carcinogens, toxins, and reactive oxygen species (ROS), that are recognized to cause oxidative stress and trigger inflammatory responses in various biological systems [36]. Studies have shown that exposure to CSE upregulates the production of pro-inflammatory cytokines, chemokines, and adhesion molecules in lung epithelial cells and immune cells [37]. Additionally, COPD is one of the respiratory disorders whose pathophysiology is linked to inflammation generated by CSE. Research by Zong D et al. reveals that CSE exacerbates inflammation in COPD by upregulating lncRNA CCAT1, which activates the ERK signaling pathway through miR-152-3p sponging in human bronchial epithelial cells [38]. Additionally, another study of Zong D demonstrates that CSE induces endothelial apoptosis in COPD by modulating Notch signaling, with Notch1 attenuating apoptosis via ERK signaling pathway inhibition [39]. Moreover, Zhu X et al. showed that CSE would aggravate the inflammatory damage of human bronchial epithelial cells, but the overexpression of *SRY-Related HMG-Box 9* (*SOX9*) would inhibit the expression of *Stromal Interaction Molecule 1* (*STIM1*), thus alleviating the impact [40]. Our study observed that CSE induction resulted in a concentration-dependent increase in *CD244* expression, apoptosis rate of HBE cells, and levels of inflammatory factors, including IL-6, TNF-α, and IL-8. This suggests that CSE enhances *CD244* expression and promotes HBE cell apoptosis and its inflammation, which may be related to the pathophysiology of COPD. To elucidate the functional role of *CD244* in cigarette smoke exposure, we performed in vitro cell experiments. Knockdown of *CD244* was found to significantly attenuate CSE-induced apoptosis and inflammation in HBE cells. Conversely, overexpression of *CD244* triggers the opposite response, exacerbating apoptotic and inflammatory processes. These findings highlight the contribution of *CD244* to pro-inflammatory and pro-apoptotic pathways and suggest that its overexpression amplifies the deleterious effects of cigarette smoke on lung tissue.

A protein tyrosine phosphatase that is expressed by the PTPN11 gene is called *SHP2*, or Src homology 2 domain-containing phosphatase-2 [41]. It plays a crucial role in various cellular processes, including cell growth, survival, and differentiation. In the context of apoptosis, *SHP2* is associated with both promoting and inhibiting cell death, depending on the specific cellular context and signaling pathways involved. For instance, *SHP2* can enhance cell survival by activating pro-survival signaling pathways such as the Ras-ERK pathway or inhibiting pro-apoptotic pathways like the JNK pathway [42]. Conversely, in certain circumstances, *SHP2* may also induce apoptosis by modulating the activity of apoptotic regulators or interacting with apoptotic signaling molecules [43]. In addition, research has indicated a strong correlation between *SHP2* and inflammation. Studies by Li FF et al. have revealed a correlation between elevated levels of *SHP2* and increased inflammatory markers in COPD, suggesting that selective inhibition or knockdown of *SHP2* can reduce pulmonary inflammation [44]. Additionally, research by Chang CJ et al. has demonstrated *SHP2* as a protein tyrosine phosphatase associated with chronic pulmonary inflammation and fibrosis in COPD [45]. The potential of *SHP2* as a therapeutic target for COPD is highlighted by these studies taken together.

We confirmed the physical interaction between *CD244* and *SHP2* by Co-IP analysis, supporting the notion that *SHP2* is a potential interaction partner of *CD244* in COPD. Further examination revealed that *CD244* positively regulates *SHP2* levels following exposure to CSE. Our study also explored the functional consequences of *CD244* overexpression and *SHP2* knockdown in CSE-treated HBE cells. *CD244* overexpression promoted CSE-induced apoptosis and inflammation, whereas *SHP2* knockdown effectively reversed these effects. These findings suggest that *CD244* may promote apoptosis and inflammation in COPD through the upregulation of *SHP2*, providing new insights into the pathogenesis of COPD. We further investigated the potential involvement of NF-κB and MAPK signaling pathways in *CD244*-mediated effects. Previous studies have demonstrated that dysregulation of these pathways promotes the recruitment of inflammatory cells and cytokine production, contributing to the pathogenesis of various lung diseases, including COPD. For example, activation of the MAPK pathway is directly linked to the inflammatory response in asthma, another chronic lung disease, leading to bronchial hyperresponsiveness and inflammatory cell infiltration [46]. Similarly, in idiopathic pulmonary fibrosis, aberrant NF-κB signaling is associated with fibroblast activation and excessive tissue remodeling [47]. In the context of COPD, cigarette smoke as a major etiology triggers the activation of NF-κB and MAPK signaling pathways, leading to the release of pro-inflammatory cytokines and chemokines, thereby perpetuating the inflammatory cycle and tissue damage [48]. Our results further confirmed this, and the regulation of MAPK and NF-κB signaling pathways by *CD244* and *SHP2* in CSE-treated HBE cells showed that *CD244* amplified pathway activation and promoted the dynamic interaction of inflammation and apoptosis. In contrast, knockdown of *SHP2* counteracted the effects of *CD244*, suggesting that it may serve as a regulatory mechanism to reduce COPD-related inflammatory responses. Further studies using the MAPK inhibitor SB203580 confirmed that *CD244* and *SHP2* act through the MAPK and NF-κB signalling pathways in the regulation of CSE-treated HBE cells. Our findings highlight the relevance of CSE-treated HBE cell models in studying COPD pathogenesis. In particular, the interaction of *CD244*/*SHP2* and MAPK signalling pathways provides new insights into understanding the inflammatory and apoptotic processes in COPD.

This study provides insight into the interaction between *CD244* and *SHP2* in COPD and how they affect the inflammatory response by regulating the MAPK/NF-κB signalling pathway. We found that *CD244* expression was regulated by CSE concentration and was dose-dependently related to the levels of inflammatory cytokines (IL-6, IL-8, TNF-α). Furthermore, the physical interaction between *CD244* and *SHP2* plays a key regulatory role in CSE-induced inflammatory and apoptotic responses in HBE cells.The MAPK signalling pathway plays a central role in regulating cellular responses to various stimuli, including growth factors, stress and inflammation. In the context of COPD, activation of the MAPK signalling pathway is closely associated with the production of inflammatory cytokines. Our study showed that knockdown of *CD244* reduced the phosphorylation levels of p-JNK, p-ERK, and p-P38 in CSE-induced HBE cells, suggesting that *CD244* may contribute to the inflammatory response by activating the MAPK pathway. Whereas *CD244* overexpression exacerbated these effects, knockdown of *SHP2* was able to reverse the inflammatory and apoptotic responses induced by *CD244* overexpression.

These findings highlight the important role of the MAPK signalling pathway in the pathogenesis of COPD and suggest that targeting the MAPK signalling pathway may provide novel therapeutic strategies for COPD treatment.MAPK pathway inhibitors, such as SB203580, have been shown to attenuate CSE-induced inflammation and apoptosis in HBE cells, supporting the critical role of the MAPK pathway in the inflammatory process of COPD. The use of MAPK pathway inhibitors may help to reduce inflammation and tissue damage in COPD patients, thereby improving disease prognosis. In addition, our study suggests that *CD244* and *SHP2* may interact via MAPK and NF-κB signalling pathways to co-regulate inflammatory and apoptotic responses in COPD.

Considering the key role of *CD244* in the inflammatory process of COPD, the development of small molecule drugs or biologics that can specifically inhibit the activity of *CD244* may provide new therapeutic options for COPD treatment. The use of RNA interference technology to reduce *CD244* expression has shown potential to attenuate CSE-induced inflammation and apoptosis in in vitro experiments. We also propose the use of immunomodulatory therapies such as monoclonal antibodies to block the interaction of *CD244* with its ligands, which may help to attenuate the inflammatory response in COPD patients. More importantly, our study suggests that co-targeting *CD244* and *SHP2* may have a synergistic effect, offering the possibility of new combination therapies for COPD treatment. In addition, by gaining insights into the expression and function of *CD244* in different COPD patients, we expect to be able to provide patients with personalised therapeutic regimens and achieve precision therapy.

Although our study provided valuable insights, we recognise that there are some limitations that need to be further explored and addressed in future studies. Firstly, our study was largely based on in vitro experiments using HBE as a model to mimic the pathological environment of COPD. This model is very helpful for understanding molecular mechanisms and disease processes, but it cannot fully replicate the complex physiological and pathological conditions in the human body. Therefore, our results need to be validated in an in vivo model to determine the exact role of *CD244* and *SHP2* in vivo. Second, although we used multiple biological replicates to ensure the reliability of our experimental results, the sample size was limited, which may limit the generalisability and extrapolation of our findings. Future studies should be considered in larger sample sizes and in different populations to validate our findings and explore the effects of different genetic backgrounds and environmental factors on *CD244* and *SHP2* expression. In addition, our study mainly focused on the regulatory role of *CD244* and *SHP2* on the MAPK/NF-κB signalling pathway. However, the pathogenesis of COPD is complex and involves multiple signalling pathways and molecular mediators. Therefore, future studies need to explore other signalling pathways that may be involved in the pathogenesis of COPD and the potential mechanisms by which they interact with *CD244* and *SHP2*. At the technical level, we faced some challenges, such as the inability to directly observe the localisation of Bax on the mitochondrial membrane, which limited our comprehensive understanding of the mechanism of Bax action during CSE-induced apoptosis. Due to technical and resource limitations, we were unable to employ advanced techniques such as immunofluorescence or subcellular fractionation to precisely localise Bax protein. In addition, we were unable to accurately measure the ratio of Bax to Bcl-2, which limited our in-depth analysis of the apoptotic signalling balance. Future studies will need to address these technical difficulties to gain a more comprehensive understanding of the molecular mechanisms underlying the effects of CSE on apoptosis in HBE cells. Finally, our study did not directly assess the expression and function of *CD244* and SHP2 in clinical samples, which limits our ability to directly link experimental results to clinical relevance. Future studies should include analysis of clinical samples from COPD patients to determine the potential of *CD244* and *SHP2* as potential biomarkers and therapeutic targets. In conclusion, despite these limitations of our study, we believe that our findings provide new perspectives for understanding the molecular mechanisms of COPD and lay the foundation for future research and development of therapeutic strategies. We look forward to collaborating with our peers to further explore the roles of *CD244* and *SHP2* in COPD and to overcome these limitations.

## Conclusion

In this study, we elucidated the critical roles of *CD244* and *SHP2* in HBE cell apoptosis and inflammation produced by CSE. *In vitro* experiments confirmed that overexpression of *CD244* enhanced HBE cell apoptosis and inflammation produced by CSE. This effect was significantly reversed after *SHP2* knockdown, emphasizing the regulatory role of *SHP2* on the pro-inflammatory and pro-apoptotic activities of *CD244*. In addition, we demonstrated that there is an interaction between *CD244* and *SHP2*, and the CSE-treated HBE cell model provides a valuable tool to study the pathomechanisms of COPD, and targeting the *CD244-SHP2* axis and the MAPK/NF-κB signalling pathway could provide novel therapeutic approaches to reduce inflammation in COPD and provide disease management and treatment with New strategies.

## Supporting information

S1 File (7Z)

S2 File (7Z)

S3 File (7Z)

S4 File (7Z)

S5 File (7Z)

S6 File (7Z)

S7 File (7Z)

S8 File (7Z)

S9 File (7Z)

S10 File (7Z)
